# Comparison of Biologic Disease-Modifying Antirheumatic Drug Therapy Persistence Between Biologics Among Rheumatoid Arthritis Patients Switching from Another Biologic

**DOI:** 10.1007/s40744-014-0006-3

**Published:** 2014-12-23

**Authors:** Stephen S. Johnston, Donna McMorrow, Amanda M. Farr, Paul Juneau, Sarika Ogale

**Affiliations:** 1 Truven Health Analytics, 7700 Old Georgetown Rd, Ste 650, Bethesda, MD 20814 USA; 2 Genentech, Inc, 1 DNA Way, South San Francisco, CA 94080 USA

**Keywords:** Biologics, Persistence, Rheumatoid arthritis, Switching

## Abstract

**Introduction:**

To compare biologic disease-modifying antirheumatic drug therapy persistence between biologics among patients with rheumatoid arthritis (RA) who previously used ≥1 other biologic.

**Methods:**

Using a large United States administrative claims dataset, we identified adult patients with RA initiating abatacept, adalimumab, certolizumab, etanercept, golimumab, infliximab, or tocilizumab between January 1, 2010 and January 1, 2012 (initiation date = index). Patients were required to have used ≥1 other biologic before index. Outcomes were biologic persistence, defined in two alternative ways: (1) time from initiation until switching to a different biologic (time to switch) and (2) time from initiation until switching or the first occurrence of a 90-day gap in treatment with the initiated biologic (time to switch/discontinuation). Rituximab was excluded from analyses due to retreatment based on clinical evaluation, which complicates the measurement of persistence. Multivariable survival analyses compared persistence outcomes between tocilizumab and the other biologics, adjusting for patient characteristics.

**Results:**

The sample comprised 9,782 biologic initiations; mean age 54 years and 82% female. Compared with tocilizumab, the hazards of switching biologic therapy were significantly higher for abatacept [hazard ratio (HR) = 1.19, *P* = 0.041], adalimumab (HR = 1.39, *P* < 0.001), certolizumab (HR = 1.39, *P* < 0.001), golimumab (HR = 1.20, *P* = 0.047), and infliximab (HR = 1.33, *P* < 0.001), but not significantly different for etanercept (HR = 1.19, *P* = 0.095); the hazards of switching/discontinuing biologic therapy were significantly higher for adalimumab (HR = 1.16, *P* = 0.014) and certolizumab (HR = 1.15, *P* < 0.012), but not significantly different for abatacept (HR = 1.08, *P* = 0.229), etanercept (HR = 0.97, *P* = 0.644), golimumab (HR = 0.99, *P* = 0.829), and infliximab (HR = 0.97, *P* = 0.721).

**Conclusions:**

This is one of the first studies of biologic persistence to focus specifically on patients with RA who are not naïve to biologic treatment. Among patients with RA who previously used ≥1 other biologic, tocilizumab-treated patients had similar or significantly better biologic persistence compared with other biologics.

**Electronic supplementary material:**

The online version of this article (doi:10.1007/s40744-014-0006-3) contains supplementary material, which is available to authorized users.

## Introduction

The 2010 European League Against Rheumatism (EULAR) and 2012 American College of Rheumatology (ACR) recommendations on rheumatoid arthritis (RA) management suggest switching to a different disease-modifying antirheumatic drug (DMARD) when biologic-treated patients experience treatment failure, lack of efficacy, or toxicity [1, 2]. Accordingly, a switch between biologics may signal that the therapy from which a patient has switched was ultimately suboptimal for that patient; indeed, lack of efficacy and adverse events are among the most commonly documented reasons for switching biologic therapies [3–5].

Very little information has been published regarding biologic therapy persistence across biologic agents in the real-world setting and comparative information on biologic persistence for certolizumab, golimumab, and tocilizumab is unavailable. Furthermore, there is little information on biologic persistence among patients with RA who are not naïve to biologic treatment. Thus, the objective of this retrospective, observational cohort study was to compare biologic therapy persistence between biologics among patients with RA who have previously used at least one other biologic.

## Methods

### Overview of Study Design

This was a retrospective, observational cohort study based on administrative claims data. The sample comprised patients with RA initiating biologic therapy after previously using at least one other biologic agent. Study outcomes were biologic persistence, defined in two alternative ways: (1) time from initiation until switching to a different biologic (time to switch) and (2) time from initiation until switching or the first occurrence of a 90-day gap in treatment with the initiated biologic (time to switch/discontinuation).

### Data and Setting

This study’s data were administrative claims data extracted from the Truven Health MarketScan^®^ (Truven Health, Ann Arbor, MI, USA) Commercial Claims and Encounters (Commercial) and Medicare Supplemental and Coordination of Benefits (Medicare Supplemental) databases. These databases represent a non-probability sample and comprise enrollment information and inpatient medical, outpatient medical, and outpatient pharmacy claims data for individuals with employer-sponsored primary or Medicare supplemental health insurance. No patients in these databases are covered under Medicaid insurance. In 2011 alone, the study databases contained data for over 40 million unique individuals. These databases have been used in multiple published epidemiologic evaluations related to RA [6].

The study databases satisfy the conditions set forth in Sections 164.514 (a)–(b)1ii of the Health Insurance Portability and Accountability Act of 1996 privacy rule regarding the determination and documentation of statistically de-identified data. Because this study used only de-identified patient records and does not involve the collection, use, or transmittal of individually identifiable data, Institutional Review Board approval to conduct this study was not necessary.

As described in greater detail below, study variables were measured from the database using enrollment records, International Classification of Diseases, 9th Revision, Clinical Modification (ICD-9-CM) codes, Current Procedural Technology 4th edition (CPT-4^®^) codes, Healthcare Common Procedure Coding System (HCPCS) codes, and National Drug Codes (NDCs), as appropriate [7].

### Patient Selection Criteria

Patients included for study were patients with RA initiating a biologic treatment after previously using ≥1 other biologic. As described below, patients were classified as having RA on the basis of ICD-9-CM codes recorded on their medical claims and exposure to biologic therapy was identified on the basis of a prescription fill or visit to a physician during which an infusion was administered. Specifically, patients were included in the analysis if they met all of the following selection criteria: initiated a biologic agent (abatacept, adalimumab, certolizumab, etanercept, golimumab, infliximab, or tocilizumab) between January 1, 2010 and January 1, 2012 (the dates of initiation for biologic agents used during this period were designated as the index dates); used at least one other biologic at any time prior to the index date (i.e., were not biologic naïve); had at least one non-diagnostic medical claim (i.e., excluding medical claims such as radiology and venipuncture, which may represent services that are used to diagnose or rule out the presence of a condition) with a diagnosis of RA (ICD-9-CM code 714.0x) between January 1, 2009 and March 31, 2012; were aged 18 years or older on the index date; were continuously enrolled for at least 6 months pre-index (designated as the baseline period) and 3 months post-index; and had no medical claims with diagnosis codes for any non-RA indication of biologic agents (ankylosing spondylitis, chronic lymphocytic leukemia, Crohn’s disease, juvenile idiopathic arthritis, polyarteritis nodosa, non-Hodgkin’s lymphoma, plaque psoriasis, psoriatic arthritis, ulcerative colitis, or Wegener’s granulomatosis) within the baseline period. As described in greater detail below, rituximab was excluded from analyses due to retreatment based on clinical evaluation, which complicates the measurement of persistence.

An episode-based study design was used wherein patients were allowed to contribute multiple observations to the dataset, one for each biologic they initiated sequentially during the study period. Thus, patients were followed forward in time after their first qualifying biologic initiation to capture all subsequent episodes of biologic use. Episodes of biologic use began with initiation of a new biologic and ended with switch to a different biologic, the end of the study period (March 31, 2012), or insurance disenrollment.

### Biologic Therapy Persistence Outcomes

The study outcomes were biologic therapy persistence, defined in two alternative ways: (1) time from initiation until switching to a different biologic (time to switch) and (2) time from initiation until switching or the first occurrence of a 90-day gap in treatment with the initiated biologic. The follow-up of patients, who did not experience a switch, was censored at the end of the study period (March 31, 2012) or insurance disenrollment. As noted above, rituximab was excluded from the analyses. This is because courses of rituximab may be given every 24 weeks or based on clinical evaluation, we could not define a single time point from which a 90-day gap in therapy exposure would begin. Furthermore, because the re-treatment interval for rituximab is no sooner than 4 months after the prior infusion, it is possible that physicians would wait longer to switch patients from RTX, as compared with other biologics that have shorter re-treatment intervals. Thus, we conservatively chose to exclude rituximab from the analyses due to the uniqueness of re-treatment, which can complicate the measurement of persistence. Rituximab use was still tracked, however, for the purpose of identifying cases in which patients switched to rituximab.

### Covariates

The study covariates included patient demographics and clinical characteristics thought to potentially confound the relationship between the persistence outcomes and biologic agent. Patient demographics were measured at index and are listed in Table 1. Patient clinical characteristics were measured throughout the baseline period and are listed in Table 2 [8, 9]. Included in the list of clinical characteristics was an administrative claims-based index for RA severity (CIRAS) score, which has been shown to have moderate correlations with a previously validated records-based index of severity that has established construct validity and convergent validity with the Disease Activity Score (DAS28) [10]. The CIRAS assigns a numerical value based on orders for inflammatory markers, number of platelet counts and chemistry panels ordered, rheumatoid factor, rehabilitation visits, age and gender, presence of Felty’s syndrome and number of rheumatology visits. Details on the algorithm can be found in Ting et al. [10]. These covariates are consistent with prior research showing that demographic factors as well as measures of comorbidity, medication and other healthcare resource use to predict time to biologic discontinuation [11].

**Table 1:** Patient demographics measured at index

Demographic	TCZ	ABA	INF	ADA	CZP	ETA	GOL
	*N* = 1,090	*N* = 1,759	*N* = 922	*N* = 2,179	*N* = 962	*N* = 1,675	*N* = 1,195
Age, mean (±SD)	54.7 (±12.3)	55.4 (±12.8)	53.5 (±13.2)	52.9 (±12.9)	52.9 (±12.0)	52.8 (±12.6)	52.5 (±12.4)
Female, %	83.1%	83.2%	81.2%	81.0%	80.1%	80.7%	82.3%
Geographic region, %							
Northeast	12.3%	11.8%	13.3%	14.2%	13.8%	13.3%	11.1%
North Central	27.3%	28.1%	22.2%	25.0%	22.0%	26.3%	27.6%
South	39.0%	42.0%	43.5%	37.2%	44.5%	40.6%	40.1%
West	20.6%	17.1%	20.0%	22.9%	19.1%	19.0%	20.7%
Unknown	0.7%	1.0%	1.0%	0.7%	0.5%	0.8%	0.5%
Insurance plan type, %							
Comprehensive	11.0%	13.8%	12.4%	10.1%	8.5%	10.0%	10.1%
EPO	1.7%	1.9%	1.3%	1.7%	2.4%	1.7%	1.7%
HMO	15.2%	14.1%	16.7%	16.4%	12.8%	14.1%	12.6%
Point of service	7.9%	8.2%	6.9%	7.7%	7.7%	8.1%	10.4%
PPO	53.9%	53.2%	55.0%	55.6%	58.2%	55.9%	57.6%
POS with capitation	0.9%	0.9%	0.8%	0.6%	1.2%	0.5%	0.8%
CDHP	3.7%	2.9%	2.4%	3.7%	4.3%	4.8%	3.8%
HDHP	1.7%	1.8%	1.6%	1.9%	0.9%	1.7%	0.8%
Unknown	4.0%	3.2%	2.9%	2.5%	4.0%	3.0%	2.3%
Population density, %							
Urban	84.9%	85.2%	83.8%	83.8%	85.9%	81.5%	82.8%
Rural	14.4%	13.8%	15.2%	15.6%	13.6%	17.8%	16.8%
Unknown	0.7%	1.0%	1.0%	0.7%	0.5%	0.7%	0.3%
Year of index, %							
2010	33.4%	48.9%	47.7%	45.4%	46.3%	43.2%	60.2%
2011	66.6%	51.1%	52.3%	54.6%	53.7%	56.8%	39.8%

*ABA* abatacept, *ADA* adalimumab, *CDHP* Consumer Directed Health Plan, *CZP* certolizumab, *EPO* exclusive provider organization, *ETA* etanercept, *GOL* golimumab, *HDHP* High Deductible Health Plan, *HMO* Health Maintenance Organization, *INF* infliximab, *POS* point of service, *PPO* preferred provider organization, *SD* standard deviation, *TCZ* tocilizumab

**Table 2:** Patient clinical characteristics measured during 6-month (pre-index) baseline period

Clinical characteristic	TCZ	ABA	INF	ADA	CZP	ETA	GOL
	*N* = 1,090	*N* = 1,759	*N* = 922	*N* = 2,179	*N* = 962	*N* = 1,675	*N* = 1,195
CIRAS, mean (±SD)	3.6 (±0.9)	3.5 (±1.0)	3.6 (±1.1)	3.6 (±0.9)	3.6 (±0.9)	3.7 (±1.2)	3.6 (±0.9)
NSAIDs, %	38.5%	39.3%	41.9%	41.8%	45.2%	42.8%	41.7%
Corticosteroids, %	80.8%	78.6%	76.7%	71.1%	74.3%	72.4%	69.5%
Analgesics, %	63.9%	58.6%	56.9%	52.6%	56.3%	58.0%	53.7%
Non-biologic DMARDs, mean (±SD)	0.9 (±0.7)	0.9 (±0.7)	1.0 (±0.7)	0.9 (±0.7)	0.9 (±0.7)	0.9 (±0.7)	0.9 (±0.7)
Extraarticular disease^a^, %	5.5%	4.5%	3.4%	4.2%	4.2%	3.2%	4.1%
DCI, mean (±SD)	1.4 (±0.9)	1.4 (±0.9)	1.4 (±0.9)	1.2 (±0.8)	1.3 (±0.8)	1.3 (±0.8)	1.3 (±0.9)
Number of unique 3-digitICD-9-CM, mean (±SD)	21.7 (±16.6)	18.8 (±14.4)	18.7 (±14.6)	15.5 (±13.0)	18.6 (±14.9)	16.8 (±13.7)	17.5 (±14.8)
Number of unique NDCs, mean (±SD)	21.2 (±15.7)	17.8 (±12.3)	18.5 (±13.6)	15.3 (±10.9)	19.5 (±14.4)	16.8 (±11.9)	18.3 (±14.6)
Immediately prior drug = anti-TNF, %	49.5%	85.8%	72.5%	91.2%	85.1%	88.2%	87.3%

*ABA* abatacept, *ADA* adalimumab, *CIRAS* claims-based index for rheumatoid arthritis severity, *CZP* certolizumab, *DCI* Deyo-Charlson comorbidity index, *DMARD* disease-modifying antirheumatic drug, *ETA* etanercept, *GOL* golimumab, *ICD*-*9*-*CM* International Classification of Diseases, 9th Revision, Clinical Modification, *INF* infliximab, *NDC* National Drug Code, *NSAIDs* non-steroidal anti-inflammatory drugs, *SD* standard deviation, *TCZ* tocilizumab, *TNF* tumor necrosis factor-α

^a^Rheumatoid nodules, Sjögren’s syndrome, retinal vasculitis, other vasculitis, Felty’s syndrome, or rheumatoid lung

### Statistical Analysis

Bivariate analyses were used to display summary statistics for the variable distributions, stratified by biologic agent. The Kaplan–Meier (or product-limit) method was used to estimate the unadjusted probabilities of the persistence outcomes at 1 and 2 years after initiation [12]. Multivariable Cox proportional hazards models with the Huber-White “sandwich” variance estimator—which accounted for the possibility of multiple observations per patient—were used to compare the persistence outcomes between the biologic agents, adjusting for patient demographics and clinical characteristics listed in Tables 1 and 2 [13–15]. The variance inflation factor was used to assess multi-collinearity of the model’s independent variables [16]. Plots of Schoenfeld residuals were used to assess whether the model’s independent variables met the proportionality assumption of the Cox proportional hazards modeling approach [17]. In the multivariable analyses, tocilizumab was chosen as the reference category because for the time period during which this study was conducted, tocilizumab was the last entrant to the market and, among the more recently approved biologics including certolizumab and golimumab, had a unique (non-anti-TNF) mechanism of action. The choice of tocilizumab as the reference category therefore provided comparative information between it and each of the other available biologics. All analyses were performed using SAS version 9.2 (Cary, NC, USA). *P* values <0.05 were considered, a priori, to be statistically significant.

## Results

### Patient Characteristics

From among 360,508 patients with at least one non-diagnostic medical claim (i.e., excluding medical claims such as radiology and venipuncture, which may represent services that are used to diagnose or rule out the presence of a condition) for RA between January 1, 2009 and March 31, 2012, a total of 16,999 initiations of biologic therapy after use of at least one prior biologic were identified. From among these 16,999 initiations, 138 were excluded because they did not meet the study’s age criteria, an additional 3,063 were excluded because they did not meet the study’s continuous enrollment criteria, an additional 3,106 were excluded because they had at least one medical claim with a diagnosis code for a non-RA indication of biologic therapy, and 910 were excluded because they were rituximab initiations. The final sample comprised 9,782 biologic initiations.

Tables 1 and 2 display patients’ demographics and baseline clinical characteristics, respectively, stratified by biologic agent. The average patient age ranged from 52.5 years in golimumab-treated patients to 55.4 in abatacept-treated patients. The proportion of females ranged from 80.1% in certolizumab-treated patients to 83.2% in abatacept-treated patients. Across all biologics, the average number of non-biologic DMARDs used prior to initiation was approximately one. Use of corticosteroids prior to initiation was common, with proportions ranging from 69.5% in golimumab-treated patients to 80.8% in tocilizumab-treated patients.

### Biologic Therapy Persistence

Table 3 displays probabilities of biologic therapy persistence, as defined by time to switch, at 1 and 2 years after initiation, unadjusted for demographic or clinical characteristics. A total of 2,553 switches to a different biologic were observed. At 1 year after initiation, the probability of persisting on therapy without switching ranged from 68.8% in certolizumab-treated patients to 76.6% in tocilizumab-treated patients; at 2 years after initiation, these probabilities ranged from 52.4% in certolizumab-treated patients to 66.9% in etanercept-treated patients.

**Table 3:** Unadjusted probabilities of biologic DMARD therapy persistence (time to switch to different biologic DMARD) at 1 and 2 years after initiation

Follow-up and persistence	TCZ	ABA	INF	ADA	CZP	ETA	GOL
	*N* = 1,090	*N* = 1,759	*N* = 922	*N* = 2,179	*N* = 962	*N* = 1,675	*N* = 1,195
Median days of follow-up overall^a^	317	361	358	346	344	338	431
Median days of follow-up until event^b^	252	281	267	263	261	265	299
*N* switching to different biologic DMARD	238	449	257	580	291	384	354
Unadjusted probability of biologic DMARD therapy persistence, %^c^							
1 year after initiation	76.6	73.5	72.5	70.9	68.8	75.7	70.9
2 years after initiation	60.6	58.8	55.8	60.6	52.4	66.9	58.5

*ABA* abatacept, *ADA* adalimumab, *CZP* certolizumab, *DMARD* disease-modifying antirheumatic drug, *ETA* etanercept, *GOL* golimumab, *INF* infliximab, *TCZ* tocilizumab

^a^Days from initiation until disenrollment or March 31, 2012

^b^Days from initiation to switch to a different biologic DMARD or censoring at disenrollment or March 31, 2012

^c^Kaplan–Meier estimate

**Fig. 1:**
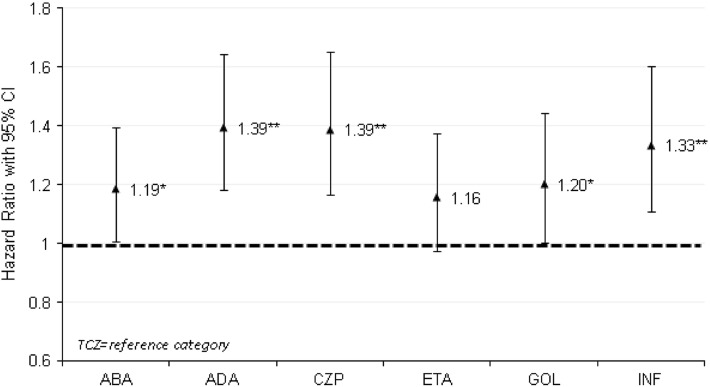
Multivariable-adjusted hazard ratios (HRs) for time to non-persistence with biologic therapy (time to switch to different biologic DMARD), treating TCZ as reference category. See Appendix in the Electronic Supplementary Material for full multivariable analysis results. *P < 0.05 vs. TCZ, **P < 0.01 vs. TCZ. *ABA* abatacept, *ADA* adalimumab, *CI* confidence interval, *CZP* certolizumab, *DMARD* disease-modifying antirheumatic drug, *ETA* etanercept, *GOL* golimumab, *INF* infliximab, *TCZ* tocilizumab

Figure 1 displays the multivariable-adjusted hazard ratios (HRs) for time to switch, treating tocilizumab as reference category. Compared with tocilizumab, the hazards of switching biologic therapy were significantly higher for abatacept (HR = 1.19, *P* = 0.041), adalimumab (HR = 1.39, *P* < 0.001), certolizumab (HR = 1.39, *P* < 0.001), golimumab (HR = 1.20, *P* = 0.047), and infliximab (HR = 1.33, *P* < 0.001), but not significantly different for etanercept (HR = 1.16, *P* = 0.095).

Table 4 displays probabilities of biologic therapy persistence, as defined by time to switch/discontinuation, at 1 and 2 years after initiation, unadjusted for demographic or clinical characteristics. A total of 2,046 switches to a different biologic and 2,490 discontinuations were observed. At 1 year after initiation, the probability of persisting on therapy without switching or discontinuing ranged from 45.5% in certolizumab-treated patients to 53.3% in infliximab-treated patients; at 2 years after initiation, these probabilities ranged from 29.0% in certolizumab-treated patients to 42.4% in etanercept-treated patients.

**Table 4:** Unadjusted probabilities of biologic DMARD therapy persistence (time to time to switch to different biologic DMARD/discontinuation of the initiated biologic DMARD) at 1 and 2 years after initiation

Follow-up and persistence	TCZ	ABA	INF	ADA	CZP	ETA	GOL
	*N* = 1,090	*N* = 1,759	*N* = 922	*N* = 2,179	*N* = 962	*N* = 1,675	*N* = 1,195
Median days of follow-up overall^a^	317	361	358	346	344	338	431
Median days of follow-up until event^b^	172	195	203	176	180	186	199
*N* switching to different biologic DMARD	178	354	208	470	224	328	284
*N* discontinuing initiated biologic DMARD^c^	286	498	196	571	258	377	304
Unadjusted probability of biologic DMARD therapy persistence, %^d^							
1 year after initiation	51.5	46.9	53.3	46.3	45.4	53.1	47.7
2 years after initiation	38.8	31.9	35.9	36.4	29.0	42.4	36.4

*ABA* abatacept, *ADA* adalimumab, *CZP* certolizumab, *DMARD* disease-modifying antirheumatic drug, *ETA* etanercept, *GOL* golimumab, *INF* infliximab, *TCZ* tocilizumab

^a^Days from initiation until disenrollment or March 31, 2012

^b^Days from initiation to switch to a different biologic DMARD/discontinuation of the initiated biologic DMARD or censoring at disenrollment or March 31, 2012

^c^Discontinuation is defined as a 90-day gap in therapy

^d^Kaplan–Meier estimate

**Fig. 2:**
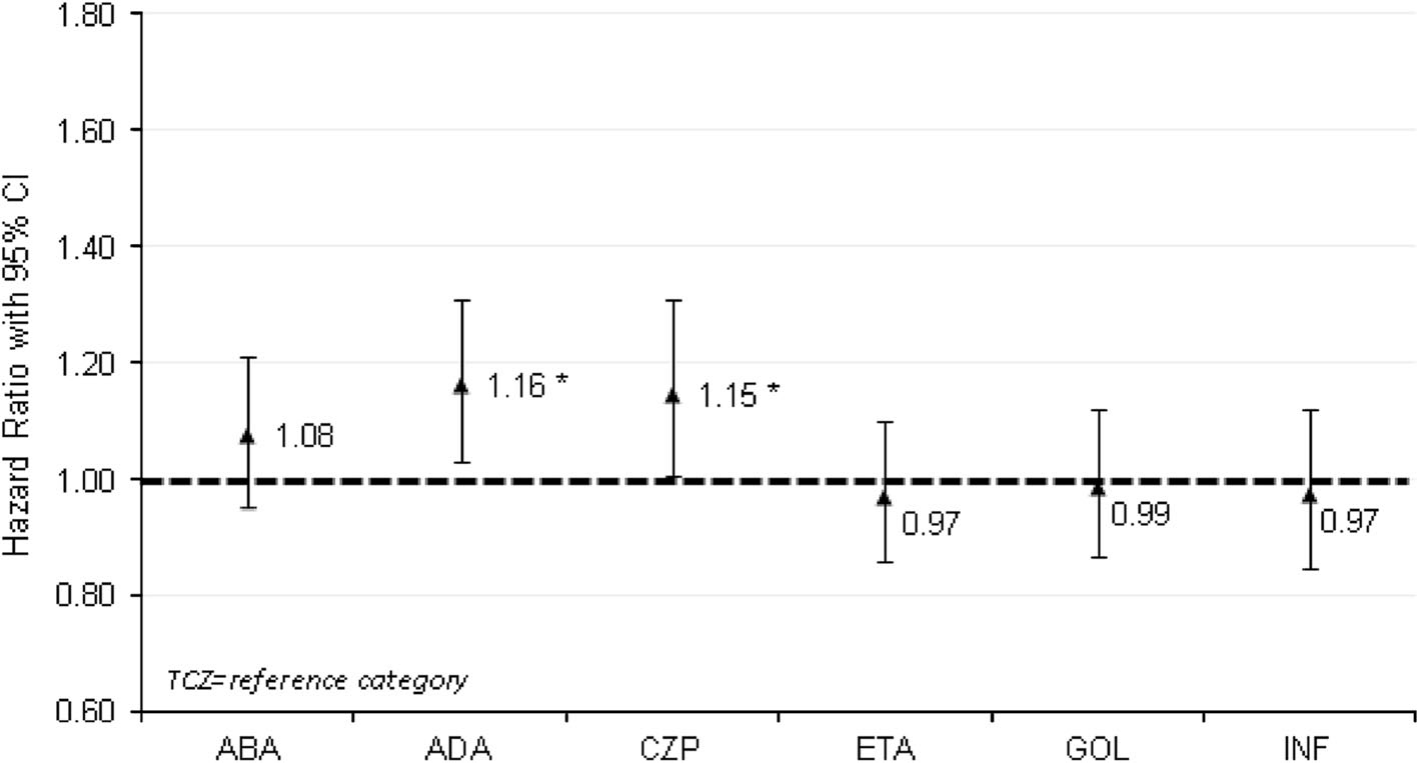
Multivariable-adjusted HRs for time to non-persistence with biologic therapy (time to time to switch to different biologic DMARD/discontinuation of the initiated biologic DMARD), treating TCZ as reference category. See Appendix in the Electronic Supplementary Material for full multivariable analysis results. *P < 0.05 vs. TCZ. *ABA* abatacept, *ADA* adalimumab, *CI* confidence interval, *CZP* certolizumab, *DMARD* disease-modifying antirheumatic drug, *ETA* etanercept, *GOL* golimumab, *INF* infliximab, *TCZ* tocilizumab

Figure 2 displays the multivariable-adjusted HRs for time to switch/discontinuation, treating tocilizumab as reference category. Compared with tocilizumab, the hazards of switching/discontinuing biologic therapy were significantly higher for adalimumab (HR = 1.16, *P* = 0.014) and certolizumab (HR = 1.15, *P* < 0.012), but not significantly different for abatacept (HR = 1.08, *P* = 0.229), etanercept (HR = 0.97, *P* = 0.644), golimumab (HR = 0.99, *P* = 0.829), and infliximab (HR = 0.97, *P* = 0.721).

## Discussion

To our knowledge, this is the first study to compare biologic therapy persistence between biologic DMARDs among patients with RA who have previously used at least one other biologic agent. Compared with tocilizumab-treated patients, the hazard of switching was significantly higher for abatacept-treated patients and anti-TNF-treated patients (except in the case of etanercept) and the hazard of switching/discontinuation was significantly higher for adalimumab-treated patients and certolizumab-treated patients. The relative trends across the comparator (non-tocilizumab) biologics were generally similar for each of the two study endpoints, with adalimumab- and certolizumab-treated patients having the highest comparative hazards of each endpoint and abatacept-, etanercept-, golimumab-, and infliximab-treated patients having relatively lower comparative hazards. While we see significantly longer time to switch for tocilizumab compared with abatacept, golimumab and infliximab, there is no difference between tocilizumab and these agents when we look at the time to switch/discontinuation endpoint. These findings show that when we define discontinuation as a gap in therapy, the persistence for tocilizumab is similar to that of these three agents, implying that tocilizumab patients might have a longer gap in therapy before switching to a different agent. Because our data source did not contain detailed reasons as to why patients switch/discontinue, we cannot conclude with certainty as to why this happens.

Owing to the uniqueness of this investigation, there are very few studies to which these results can be compared. The majority of studies examining biologic therapy persistence rates among RA patients have focused largely on the first-line setting or have included only the anti-TNF agents such as adalimumab, etanercept, and infliximab [18–22]. One prior study by Ogale et al. [11] described switching between biologics among RA patients treated with abatacept, adalimumab, etanercept, infliximab, or rituximab in first- or subsequent-line settings. Ogale et al. [11] reported that in the subsequent-line setting, adalimumab-treated patients had the highest unadjusted rates of switching to a different biologic (38.2%). The present study’s findings were similar to those of Ogale et al. [11], with unadjusted rates of switching at 1 year equaling 29.1% for adalimumab-treated patients. Furthermore, unadjusted rates of switching at 1 year for abatacept were similar between the two studies, with Ogale et al. [11] reporting 23.4% and the present study finding 26.5%. With the inclusion of certolizumab, golimumab, and tocilizumab, the present study contributes uniquely to the body of research examining biologic therapy persistence.

Among the covariates included in the multivariable models, there were several significant predictors of persistence. Predictors with consistent direction and significance across the two models included age (increase associated with better persistence), the Deyo-Charlson comorbidity index [increase (indicative of greater comorbidity) associated with better persistence], the number of non-biologic DMARDs in the baseline period (increase associated with better persistence), the number of unique three-digit ICD-9-CM diagnoses in baseline period (increase associated with slightly worse persistence), and the number of unique NDCs in the baseline period (increase associated with slightly worse persistence). With few exceptions, other covariates were generally consistent in direction across the models and statistically in significant. Although baseline use of corticosteroids was not statistically significantly associated with persistence, the high baseline use rates of corticosteroids were nevertheless notable, ranging from 69.5% in golimumab-treated patients to 80.8% in tocilizumab-treated patients. These rates were similar to the rates previously reported by Ogale et al. [11], which among subsequent-line patients ranged from 69.9% in infliximab-treated patients to 74.9% in abatacept-treated patients. Low-dose corticosteroids therapy may be part of the treatment strategy in combination with DMARDs, though the appropriate duration of therapy is debated due to the adverse event profile of corticosteroids [1].

The 2010 EULAR and 2012 ACR recommendations on RA management suggest switching to a different DMARD when biologic-treated patients experience treatment failure, lack of efficacy, or toxicity [1, 2]. Because administrative claims data do not posses clinical information regarding reasons for switching and/or discontinuation, the present study is unable to discern the underlying causes of which differences in persistence may be indicative. However, evidence from two recently presented (in conferences) observational studies including biologic-treated patients from the United States (US) suggest that among the various reasons for switching and/or discontinuation, efficacy and tolerability/safety account for at least half of all biologic discontinuations [23, 24]. Strand et al. [23] studied 6,209 biologic-treated RA patients drawn from the US Consortium of Rheumatology Researchers of North America (CORRONA) database. They reported that among those who discontinue or switch therapy within the first year of treatment, reasons for such changes included loss of efficacy (35.8%), physician preference (27.8%), safety concerns (20.1%), patient preference (17.9%), or access to treatment (9.0%) [23]. Elkin et al. [24] studied medical charts of 176 RA patients from 8 centers in the US. These patients had discontinued an anti-TNF as their first biologic DMARD and had gone on to receive a second biologic DMARD. The reported reasons for this change were failure to maintain response (46.6%), lack of initial efficacy (22.7%), safety/tolerance (17.0%), cost, insurance, or formulary-related matters (7.4%), other or unknown (7.4%), and patient or physician preference (0.6%) [24]. Evidence from populations outside of the US has also been consistent with the findings from the two aforementioned studies [3–5]. Thus, it is plausible that the switching and discontinuation patterns observed in the present study may be indicative of undesirable clinical circumstances such as treatment failure due to lack of efficacy or adverse events.

This study was subject to limitations. Administrative claims data are not collected for research purposes and the procedure and diagnosis coding on administrative claims data is recorded by healthcare practitioners to support reimbursement. Thus, miscoded or non-coded administrative claims can result in measurement error when measuring variables that rely on such coding. Because administrative claims data do not provide detailed clinical information, we do not know why patients may have switched to a different biologic. We required that all study patients have previously used at least one other biologic. It is possible that if a patient has failed multiple biologics, a patient’s physician or the patient him- or herself may be less likely to switch to an alternative therapy. If tocilizumab is reserved for later lines of therapy, this could potentially explain the lower hazards of switching among tocilizumab-treated patients. To investigate this, we quantified the average number of observed biologics used prior to initiation of treatment for each biologic group, with the limitation that these data are left-censored. We found that the average number of observed prior biologics differed very little, by only one-tenth, across the biologic groups: tocilizumab = 1.3 prior biologics, abatacept = 1.2, infliximab = 1.3, adalimumab = 1.2, certolizumab = 1.3, etanercept = 1.2, and golimumab = 1.2. Similarly, the proportion of patients with at least three prior biologics differed little across the biologic groups: tocilizumab = 4%, abatacept = 3%, infliximab = 4%, adalimumab = 2%, certolizumab = 4%, etanercept = 2%, and golimumab = 3%.

The study databases did not include information on patients’ education or socio-economic status, which may affect their access to, and ability to pay for, biologic treatments. This limitation is tempered by the fact that all patients included in the study were required to have initiated biologic therapy and had previously used at least one other biologic. Therefore it is known that they have access to more than one biologic treatment. Furthermore, the fact that they have been treated with more than one biologic is suggestive that economic limitations may be less important for the studied population than individuals, who had alternative coverage such as Medicaid insurance. Finally, these results are not generalizable to the entire US RA population, including those who are uninsured or insured through Medicaid.

## Conclusion

Among patients with RA who previously used at least one other biologic, tocilizumab-treated patients had similar or significantly better biologic persistence compared with other biologics. Such persistence differences may be reflective of treatment failure, inadequate response, side effects, or other reasons. This study’s findings may provide insights into the comparative effectiveness of biologic agents when used in the real-world setting, specifically among patients with RA who are not naïve to biologic treatment.

## Electronic supplementary material

Below is the link to the electronic supplementary material.

Supplementary material 1 (PDF 103 kb)

Supplementary material 2 (PDF 189 kb)
