# Supplementary material for: Comparison of Biologic Disease-Modifying Antirheumatic Drug Therapy Persistence Between Biologics Among Rheumatoid Arthritis Patients Switching from Another Biologic
Source: Rheumatol Ther. 2014 Dec 23;2(1):59–71. doi: 10.1007/s40744-014-0006-3 (PMC4883249; doi:10.1007/s40744-014-0006-3)
Supplement: Supplementary file 1 — Supplementary material 1 (PDF 103 kb) [file 40744_2014_6_MOESM1_ESM.pdf]

Appendix. Multivariable analyses of biologic DMARD therapy persistence

	Time to switch to different biologic DMARD*				Time to switch to different biologic DMARD/discontinuation of initiated biologic DMARD**			
	HR	95% CI		p-value	HR	95% CI		p-value
Abatacept vs. tocilizumab	1.19	1.01	1.40	0.041	1.08	0.95	1.21	0.229
Adalimumab vs. tocilizumab	1.39	1.18	1.65	0.000	1.16	1.03	1.31	0.014
Certolizumab vs. tocilizumab	1.39	1.16	1.65	0.000	1.15	1.01	1.31	0.042
Etanercept vs. tocilizumab	1.16	0.97	1.37	0.095	0.97	0.86	1.10	0.644
Golimumab vs. tocilizumab	1.20	1.00	1.44	0.047	0.99	0.87	1.12	0.829
Infliximab vs. tocilizumab	1.33	1.11	1.60	0.002	0.97	0.85	1.12	0.722
Age	0.99	0.97	1.00	0.028	0.99	0.98	1.00	0.003
Analgesics in baseline period	0.82	0.75	0.90	<0.001	1.00	0.94	1.07	0.901
CIRAS in baseline period	0.92	0.77	1.10	0.348	0.96	0.87	1.05	0.335
Corticosteroids in baseline period	0.98	0.89	1.08	0.642	0.99	0.92	1.06	0.801
DCI in baseline period	0.87	0.82	0.92	<0.001	0.96	0.92	0.99	0.015
Extraarticular disease in baseline period ***	0.96	0.79	1.16	0.642	1.02	0.88	1.19	0.787
Geographic region: North Central vs. West/unknown	0.88	0.78	0.99	0.037	0.96	0.88	1.05	0.341
Geographic region: Northeast vs. West/unknown	0.85	0.73	0.98	0.028	0.91	0.82	1.01	0.087
Geographic region: South vs. West/unknown	0.92	0.82	1.03	0.128	1.01	0.93	1.09	0.815
Health plan type: CDHP vs. PPO	0.96	0.76	1.21	0.721	0.92	0.78	1.09	0.345
Health plan type: Comprehensive vs. PPO	1.03	0.88	1.20	0.704	1.01	0.90	1.12	0.920
Health plan type: EPO vs. PPO	1.10	0.76	1.60	0.612	1.00	0.78	1.29	0.985
Health plan type: HDHP vs. PPO	1.29	0.95	1.75	0.099	1.00	0.80	1.25	0.980
Health plan type: HMO vs. PPO	1.08	0.95	1.23	0.251	1.09	1.00	1.19	0.040
Health plan type: POS vs. PPO	1.03	0.89	1.19	0.718	1.07	0.96	1.19	0.215
Health plan type: POS w/ capitation vs. PPO	1.16	0.73	1.83	0.526	0.77	0.52	1.14	0.192
Health plan type: Unknown vs. PPO	1.11	0.89	1.40	0.358	0.96	0.81	1.15	0.671
Immediately prior drug = anti-TNF	1.19	1.06	1.34	0.004	1.08	0.99	1.18	0.066
Index Year: 2011 vs. 2010****	n/a	n/a	n/a	n/a	0.79	0.74	0.84	<0.001
Male vs. female	1.04	0.93	1.16	0.489	0.99	0.92	1.07	0.860
NSAIDs in baseline period	0.95	0.87	1.03	0.238	0.93	0.88	0.99	0.020
Number of non-biologic DMARDs in baseline period	0.93	0.88	0.99	0.015	0.92	0.88	0.96	<0.001
Number of unique 3-digit ICD-9-CM diagnoses in baseline period	1.02	1.02	1.03	<0.001	1.01	1.01	1.02	<0.001
Number of unique NDCs in baseline period	1.01	1.01	1.02	<0.001	1.01	1.01	1.01	<0.001
Urban vs. rural	1.01	0.90	1.14	0.859	1.02	0.95	1.11	0.571

ABA=abatacept; ADA=adalimumab; CDHP=Consumer Directed Health Plan; CIRAS=Claims-based index for rheumatoid arthritis severity; CZP=certolizumab; DCI=Deyo-Charlson Comorbidity Index; DMARD=Disease Modifying Antirheumatic Drug; EPO=Exclusive Provider Organization; ETA=etanercept; GOL=golimumab; HDHP=High Deductible Health Plan; HMO=Health Maintenance Organization; ICD-9-CM=International Classification of Diseases, 9th Revision, Clinical Modification; INF=infliximab; NDC=National Drug Code; NSAIDs=non-steroidal anti-inflammatory drugs; POS=Point of Service; PPO=Preferred Provider Organization; SD=standard deviation; TCZ=tocilizumab; TNF=tumor necrosis factor-α

* N of patients = 9,782; N switching to different biologic DMARD = 2,553

** N of patients = 9,782; N switching to different biologic DMARD = 2,046; N discontinuing initiated biologic DMARD = 2,490 (discontinuation is defined as a 90-day gap in therapy)

*** Rheumatoid nodules, Sjögren’s syndrome, retinal vasculitis, other vasculitis, Felty’s syndrome, or rheumatoid lung

****Index year omitted from model due to violation of proportionality assumption
