# Supplementary material for: Comparison of Biologic Disease-Modifying Antirheumatic Drug Therapy Persistence Between Biologics Among Rheumatoid Arthritis Patients Switching from Another Biologic
Source: Rheumatol Ther. 2014 Dec 23;2(1):59–71. doi: 10.1007/s40744-014-0006-3 (PMC4883249; doi:10.1007/s40744-014-0006-3)
Supplement: Supplementary file 2 — Supplementary material 2 (PDF 189 kb) [file 40744_2014_6_MOESM2_ESM.pdf]

Rheumatology and Therapy

OPEN ACCESS

PEER REVIEWED SUMMARY SLIDE

- Very little information has been published regarding biologic therapy persistence across biologic agents in the real-world setting and comparative information on biologic persistence for cetrolizumab, golimumab, and tocilizumab is unavailable.
- The objective of this retrospective, observational cohort study was to compare biologic therapy persistence between biologics among patients with rheumatoid arthritis (RA) who have previously used at least one other biologic.
- Using a large United States administrative claims dataset, we identified adult RA patients initiating abatacept, adalimumab, certolizumab, etanercept, golimumab, infliximab, or tocilizumab between January 1, 2010 and January 1, 2012 (initiation date = index).
- Outcomes were biologic persistence, defined in two alternative ways: (1) time from initiation until switching to a different biologic (time to switch); (2) time from initiation until switching or the first occurrence of a 90-day gap in treatment with the initiated biologic (time to switch/discontinuation).
- Among patients with RA who previously used ≥1 other biologic, tocilizumab-treated patients had similar or significantly better biologic persistence compared with other biologics.

This summary slide represents the opinions of the authors. Sponsorship for this study was funded by Genentech, Inc. For a full list of acknowledgments and conflicts of interest for all authors of this article, please see the full text online. Copyright © The Author(s) 2014. Creative Commons Attribution Noncommercial License (CC BY-NC).
